# Prognostic Implications of Tumor Spread Through Air Spaces in Patients with Resected Lung Adenocarcinoma

**DOI:** 10.3390/diagnostics16172747

**Published:** 2026-08-27

**Authors:** Marco Andolfi, Nicolò Scarsi, Martina Mandarano, Guido Bellezza, Renato Colella, Lucrezia Burico, Domenico Pourmolkara, Francesco Ferrante, Francesca Fratturelli, Jacopo Vannucci

**Affiliations:** 1 Department of Thoracic Surgery, Azienda Ospedaliera Santa Maria della Misericordia of Perugia, University of Perugia, 06123 Perugia, Italy; 2 Department of Medicine and Surgery, University of Perugia, 06123 Perugia, Italy; 3 Section of Hygiene, Department of Life Sciences and Public Health, Università Cattolica del Sacro Cuore, 00168 Rome, Italy; 4 Section of Anatomic Pathology and Histology, Department of Medicine and Surgery, University of Perugia, 06123 Perugia, Italy

**Keywords:** STAS, lung adenocarcinoma, prognosis, risk stratification, pulmonary resection, relapse

## Abstract

**Background:** Spread through air spaces (STAS) consists of micropapillary clusters, solid nests, and/or single neoplastic cells into the air spaces of the lung parenchyma beyond the edge of the primary tumor. To date, its prognostic role in patients with lung adenocarcinoma is crucial, resulting in an independent negative factor for both overall survival (OS) and disease-free survival (DFS). The study aims to assess the prognostic impact of STAS in patients with lung adenocarcinoma undergoing curative resection. **Methods:** A retrospective, observational single-center study enrolling consecutive patients who underwent lung resections for adenocarcinoma from January 2018 to December 2023 was performed. OS and DFS were analyzed. Univariable and multivariable Cox proportional hazard models, along with the inverse probability of treatment weight–adjusted Kaplan–Meier curves, were used to evaluate the prognostic impact of STAS. A logistic regression model was used to estimate the odds ratios and 95%-confidence intervals for clinical variables associated with the STAS status. **Results:** In total, 329 patients were enrolled, and two different groups were defined (STAS-present: 30; STAS-absent: 299). No statistically significant differences were observed in OS and DFS when comparing patients according to their STAS status. Conversely, STAS-present patients were more frequently associated with pN2-nodal involvement (22.7% vs. 77.3%, *p* = 0.022), stage IIIA disease (*p* = 0.003), and the presence of an adenocarcinoma-predominant micropapillary-pattern (*p* < 0.001). Female patients were associated with better outcomes than male patients in survival analysis, while stage IIIA disease and micropapillary-pattern emerged as statistically significant after the logistic regression. **Conclusions:** Our study provides relevant information on the relationship between STAS and clinicopathological characteristics, demonstrating its potential aggressive histological feature, due to the significant correlation with stage IIIA disease, and the adenocarcinoma micropapillary pattern. Therefore, STAS may identify a subgroup of patients in whom the prognostic implications should be further investigated.

## 1. Introduction

Despite the improvements in diagnosis, staging, and treatment, lung cancer remains the leading cause of cancer-related mortality worldwide [1,2]. Therefore, considering that cancer recurrence is the most significant negative prognostic factor for survival and occurs in 20–50% of patients who have undergone lung resection, a better risk stratification approach became mandatory. In this context, the phenomenon ‘Spread Through Air Spaces’ (STAS), first described in 2015 [3] as a distinct pattern of tumor invasion in lung cancer, characterized by the presence of micropapillary clusters, solid nests, or single neoplastic cells within the alveolar spaces beyond the edge of the primary tumor, is growing in importance [1]. Since its introduction in the World Health Organization (WHO) classification of lung tumors, STAS has emerged as a relevant histopathological feature. It is prevalent, but not exclusive, in the adenocarcinoma histotype where it has been associated with more aggressive tumor behavior [4], and its incidence, regardless of the stage, ranges from 28% to 52% [5].

Two main objective classifications of STAS have been proposed: (1) related to the distance of these cells from the edge of the primary tumor (I if < 2500 μm, II if > 2500 μm) [6]; and (2) correlated with the number of cells constituting the clusters: low STAS, 1–4 cells; and high STAS, 5 or more cells [7]. However, no clear consensus has yet been reached regarding which classification should be adopted in routine histopathological diagnostic practice; consequently, the original and more subjective definition proposed by Kadota et al. [3] is still widely used, in line with the recent International Association for the Study of Lung Cancer (IASLC) and American Society of Clinical Oncology (ASCO)-College of American Pathologists (CAP) recommendations [8,9].

To date, an increasing number of studies have indicated that STAS is an independent negative prognostic factor for both overall survival (OS) and disease-free survival (DFS), particularly distant and locoregional recurrence in early-stage lung adenocarcinoma undergoing a limited resection [3,10,11,12,13,14,15].

This clinical impact was also emphasized by the IASLC, which recently recommended STAS as a histologic descriptor in the ninth edition of the TNM classification for lung cancer [16].

However, some issues remain disputed, such as the gold standard treatment for STAS-present patients and its distribution in the different stages of adenocarcinoma.

This retrospective study aims to assess the prognostic significance of STAS in patients with lung adenocarcinoma who underwent a curative-intent surgical resection, in order to identify a histopathological feature able to predict the distant recurrence risk, enhancing the risk stratification and improving the prognostic assessment in early and advanced lung adenocarcinoma.

## 2. Material and Methods

### 2.1. Patients and Study Design

This single-institution retrospective study was conducted on patients with lung adenocarcinoma who underwent lung resection, either by traditional open technique or video-assisted thoracoscopic (VATS) approach, with curative intent between January 2018 and December 2023 at “Santa Maria della Misericordia” hospital, Perugia (Italy). The study was approved by the local ethical committee (n. 5094/25) and was conducted in accordance with the ethical standards of the Helsinki Declaration by the World Medical Association. The diagnosis of lung adenocarcinoma was always confirmed histologically. Data regarding clinical and demographic characteristics, nodal involvement, tumor size, lympho-vascular invasion, lepidic pattern, grading, pathological stage, type of surgery, and STAS status were obtained from the medical records of each patient. The study population was divided into two groups based on the presence or absence of the STAS biomarker in the resected tumor. To assess the prognostic impact, OS and DFS were analyzed. Patients were contacted by phone to update the requested data. If the patient was dead, the cohabiting relative or the attending physician was contacted.

All enrolled patients were informed by medical staff and signed informed written consent to use their anonymized data for scientific purposes. The baseline characteristics and medical history of each patient were assessed at the preoperative evaluation before the operation. The indication for the surgical treatment was defined by a dedicated multidisciplinary certified meeting with thoracic surgeons, oncologists, pulmonologists, radiologists, and pathologists. The functional assessment was performed to determine the suitability of surgical treatment and followed all relevant internationally published protocols [15].

### 2.2. Eligibility Criteria

Patients were considered eligible for inclusion in this study if they were ≥18 years of age, their American Society of Anesthesiologists (ASA) score was ≤3, and they were scheduled for elective lung resection for lung adenocarcinoma. Patients were excluded from the analysis if histology was other than lung adenocarcinoma; they had undergone neoadjuvant therapy; or their individual survival data were incomplete.

### 2.3. Histopathological Assessment of STAS

**Figure 1:**
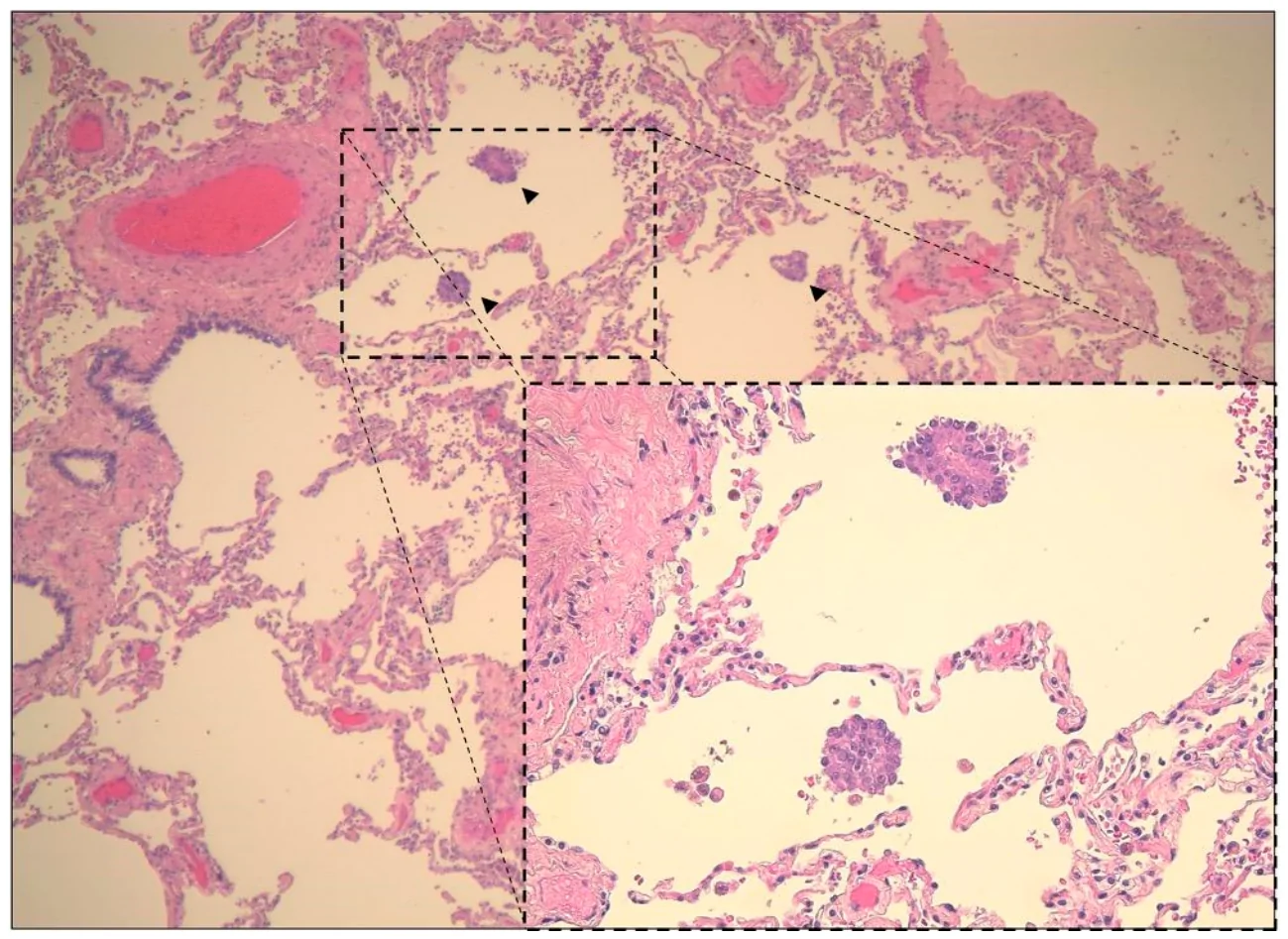
Histomorphologic appearance of multiple STAS (black arrowheads) in a Hematoxylin-and-Eosin-stained section. The inset highlights a STAS micropapillary pattern. The upper right lobectomy was performed for an adenocarcinoma with a predominant papillary pattern of growth. Original magnification: wide-field 50×, inset 200×.

Then, 4 µm-thick sections obtained from previously formalin-fixed and paraffin-embedded tissue blocks were placed on uncharged slides and subsequently stained for Haematoxylin and Eosin, using the automated Leica ST5020 Multistainer (Leica Biosystems, Nußloch, Germany) and the ST Infinity H&E Staining System kit (Leica Biosystems, Richmond, IL, USA). Two expert pathologists (G.B. and R.C.) in pleuro-pulmonary diseases assessed the presence of STAS at the time of the original diagnoses, according to the WHO classification of Thoracic Tumors in force during the study period [3,16]. Specifically, STAS was defined as neoplastic cells within airspaces in the lung parenchyma beyond the edge of the main tumor. For this purpose, when available, a specimen of macroscopically normal lung parenchyma collected at least 1 cm from the tumor bed was examined; alternatively, STAS was assessed in more than one high-power field (HPF) of normal lung parenchyma beyond the tumor margin (Figure 1).

### 2.4. Statistical Analysis

Demographic and clinicopathological data are presented as mean and standard deviation or median and range for continuous variables, and as frequency and percentage for categorical variables, as appropriate. Whether incomplete, dates were standardized and imputed, assuming interval censoring. We used descriptive statistics to calculate the incidence of STAS. Baseline comparisons of categorical data between STAS-present and STAS-absent patients were performed using Pearson’s chi-square test, if all expected cell counts were ≥5; otherwise, Fisher’s exact test was used. Variables that achieved statistical significance in the univariable analyses were included in the multivariable logistic regression model, while age and sex were included a priori regardless of their statistical significance. A logistic regression model was used to estimate odds ratios (ORs) and 95% confidence intervals (CIs) for clinical variables associated with STAS status. OS and DFS were the outcomes of interest for survival analysis. OS was defined as the interval from the date of surgical intervention to death from any cause or last follow-up in patients still alive. DFS was defined as the interval from the date of surgical intervention to the first documented recurrence or death from any cause. OS and DFS were calculated using the Kaplan–Meier method, described with the median and 95% CI, and compared using the log-rank test between patients with and without STAS. Moreover, inverse probability of treatment weight (IPTW)-adjusted Kaplan–Meier estimators were built to compare OS and DFS according to the presence of STAS. Cox proportional hazards regression models (adjusted by sex, age, and type of surgical intervention), with pairwise comparisons, were performed to estimate the hazard ratios (HRs) and the 95% CIs. Schoenfeld’s test was used to verify the proportional hazards (PH) assumption in the Cox regression model. All the tests were two-sided, and *p* values < 0.05 were considered statistically significant. Statistical analyses were performed using Stata/SE version 19.5 (StataCorp LLC, College Station, TX, USA) and R version 4.0.4 (2021-02-15; R Foundation for Statistical Computing, Vienna, Austria) through RStudio for macOS (version 2026.07.0+139.pro9).

### 2.5. IPTW-Based Propensity Score Weighting

Propensity scores were calculated as the conditional probability of STAS presence using a logistic regression model that included age, sex, and surgical group as baseline covariates to balance the two groups. The propensity score represented each patient’s estimated probability of belonging to the STAS-positive group conditional on the observed covariates. To mitigate the potential risk of confounding related to the use of a nonrandomized cohort, and to generate two groups with comparable characteristics, we performed IPTW-adjusted analyses to control for the observed differences between the compared groups [17]. In this study population, the percentage of STAS-present patients at baseline is lower than that of those without it, and we aimed to balance the groups regarding the prevalence of STAS in our sample. In the study population, the probability of being a STAS-present patient is 9.11% (i.e., a propensity score of 0.091). To balance the distribution between STAS-present and STAS-absent patients, we up-weighted each patient in the STAS-present group by taking the inverse of the propensity score. Weights were thus calculated for each individual as 1/propensity score for the STAS-present group and 1/(1 − propensity score) for the STAS-absent group, respectively. IPTW may be mathematically equivalent to standardization, as it assesses the standardized differences between groups for baseline characteristics both before and after weighting. The balance in covariates was assessed by the absolute standardized mean difference (SMD) before and after weighting. This measure was calculated to compare the difference in means between the two groups (STAS-present vs. STAS-absent) in units of standard deviation (SD), for each variable included in the propensity score (age, sex, and surgery grouped). An SMD ≤ 0.1 indicates that the covariates between the two matched groups are well-balanced. The resulting stabilized weights were subsequently incorporated into the weighted survival analyses. Propensity score weighting was performed using the teffects psmatch command in STATA software (version 19.5, StataCorp LLC, College Station, TX, USA).

## 3. Results

All results are presented in the following paragraphs in accordance with the analytical workflow of the study. The IPTW-based procedure used to achieve a covariate balance between STAS-absent and STAS-present patients is first described, with pre- and post-weighting baseline distributions reported in Table 1. Clinicopathological characteristics according to STAS status are then presented in Table 2, while detailed categories for the type of surgery, tumor size, pathological stage, and grading are reported in Supplementary Table S3. Table 3 presents the results of the multivariable binary logistic regression analysis of factors associated with the presence of STAS. Lastly, the prognostic impact of STAS is subsequently assessed for OS and DFS, both in the unweighted cohort and after an IPTW adjustment. The survival estimates at 12, 24, 36, and 60 months are reported in Table 4 and Table 5, respectively. The corresponding Kaplan–Meier curves, together with univariable Cox regression models and weighted and unweighted multivariable Cox regression analyses, are provided in the Supplementary Materials (Figures S1–S4; Tables S1 and S2).

### 3.1. IPTW-Based Propensity Score Weighting

The IPTW procedure improved the baseline covariate balance. After weighting, all absolute standardized differences were below 0.1, with an improved balance for sex (SDM before vs. after IPTW: 0.043 ⇒ 0.010) and pneumonectomy (SDM before vs. after IPTW: 0.131 ⇒ 0.011), and a borderline acceptable balance for age (SDM before vs. after IPTW: 0.151 ⇒ 0.098) and lobectomy (SDM before vs. after IPTW: 0.046 ⇒ 0.091). All results are reported in Table 1.

### 3.2. Clinicopathological Characteristics and Correlation of STAS with Clinical Variables

A final sample of 329 patients who underwent surgical intervention between January 2018 and December 2023 was included in the statistical analysis. Of these, 30 (9.1%) were classified as STAS-present and 299 (90.9%) as STAS-absent patients, respectively. Overall, the study population was almost evenly distributed by age and sex, with 169 patients (51.37%) aged <70 years (range from 38 to 85 years), and the majority (*n* = 169, 51.37%) being male. Most patients underwent a pulmonary lobectomy (71.43%) and the VATS approach (81.46%). From a pathological perspective, the majority of cases were pN0 (67.17%) and pT1 (52.58%), while early-stage disease was the most frequent stage category (57.45%). Among the histological patterns with available data, the acinar pattern was the most commonly reported (61.1%). When comparing patients according to STAS status, no statistically significant differences were observed in age, sex, type of surgery, VATS approach, tumor size, tumor stage, grading, or most histological patterns. Conversely, STAS-present patients in our sample were more frequently associated with pN2 nodal involvement (22.7% vs. 77.3% within the pN2 category, *p* = 0.022), stage IIIA disease (*p* = 0.003), and the presence of a micropapillary pattern (*p* < 0.001). Detailed clinicopathological characteristics are reported in Table 2. Concerning the logistic regression analysis, micropapillary pattern (yes versus no) [OR 18.40 (1.39–461.31), *p* = 0.0303] and IIIa stage (yes versus no) [OR 5.27 (1.15–20.90), *p* = 0.0213] were independently associated with the presence of STAS (Table 3).

### 3.3. Prognostic Implication of STAS

*(a)*  *Unweighted overall survival*

In our sample, the crude incidence rate of death was slightly higher among STAS-present patients than among STAS-absent ones (0.0066 vs. 0.0050 per person-month, or ≃ 0.50 and ≃ 0.66 per 100 person-months). However, the median OS was not reached in either group, as fewer than 50% of patients experienced the event during follow-up.

During the follow-up period, 76 deaths were observed, with 67 events in the STAS-absent group and 9 in the STAS-present group. In the unweighted Kaplan–Meier analysis, the log-rank test showed no statistically significant difference between the STAS-present and STAS-absent groups (χ^2^ = 0.66, *p* = 0.418). After univariable Cox regression analysis, with robust standard errors, STAS-present patients showed a 33% higher hazard of death than STAS-absent patients; however, this association was not statistically significant (HR: 1.33, 95% CI: 0.67–2.65, *p* = 0.415). As for the multivariable Cox regression analysis (adjusted for age (as a continuous variable), sex, and surgical intervention group), STAS-present patients showed no significant association with OS when compared to STAS-absent patients (HR: 1.37, 95% CI: 0.69–2.71, *p* = 0.372). Age was also not significantly associated with the hazard of death (HR: 1.02, 95% CI: 0.99–1.05, *p* = 0.218). By contrast, the female sex was associated with a statistically significant lower hazard of death than the male sex (HR: 0.55, 95% CI: 0.34–0.89, *p* = 0.014). Regarding the surgical group, patients in intervention group 1 (sub-lobar resections) had a significantly higher hazard of death than those in the intervention group 2 (HR: 1.64, 95% CI: 1.01–2.65, *p* = 0.044), whereas no significant difference was observed for intervention group 3 compared to group 2 (HR: 1.39, 95% CI: 0.16–12.18, *p* = 0.766). A pairwise comparison between surgical groups 1 and 3 also showed no statistically significant difference (HR: 1.18, 95% CI: 0.16–8.86, *p* = 0.872). The proportional hazards assumption was satisfied both in the univariable and multivariable analyses, as indicated by the Schoenfeld test (χ^2^ = 0.10, *p* = 0.748; χ^2^ = 2.66, *p* = 0.75).

*(b)*  *Unweighted disease-free survival*

In our sample, the crude incidence rate of the event was slightly higher among STAS-present patients than among STAS-absent patients (0.0106 vs. 0.0093 per person-month, or ≃ approximately 1.06 and ≃ approximately 0.93 per 100 person-months). However, the median DFS was reached in each group, as more than 50% of patients experienced the event during follow-up. During the follow-up period, 128 events were observed, with 114 events in the STAS-absent group and 14 in the STAS-present group. In the unweighted Kaplan–Meier analysis, the log-rank test showed no statistically significant difference between the STAS-present and STAS-absent groups (χ^2^ = 0.22, *p* = 0.639). In the univariable Cox regression analysis for DFS, STAS-present patients showed a non-significant increase in the hazard of a DFS event compared to STAS-absent patients (HR: 1.14; 95% CI: 0.65–2.00; *p* = 0.643). After adjustment for age, sex, and surgical group, the association was not statistically significant (adjusted HR: 1.15; 95% CI: 0.66–2.00; *p* = 0.619). Among the covariates included in the adjusted model, the sex was significantly associated with DFS, with female patients showing a lower hazard of a DFS event compared to male patients (HR: 0.70; 95% CI: 0.49–0.99; *p* = 0.045). Regarding the surgical group, patients in group 1 showed a higher hazard of a DFS event compared to those in intervention group 2, but this did not reach statistical significance (HR: 1.45, 95% CI: 0.98–2.14, *p* = 0.062). Similarly, patients in intervention group 3 showed a higher, but non-significant, hazard of a DFS event compared with group 2 (HR: 2.03, 95% CI: 0.80–5.14, *p* = 0.137). The pairwise comparison between surgical groups 1 and 3 also showed no statistically significant difference in the hazard of a DFS event (HR: 1.40, 95% CI: 0.53–3.66, *p* = 0.496). The proportional hazards assumption was satisfied both in univariable and multivariable DFS analyses, as indicated by the Schoenfeld test (χ^2^ = 0.03, *p* = 0.853; χ^2^ = 2.31, *p* = 0.805, respectively).

*(c)*  *Weighted overall survival*

In our sample, the weighted incidence rate of the event was 0.0049 for STAS-absent and 0.0057 for STAS-present patients (≃0.49 and 0.57 per 100 person-months). In the weighted univariable Cox regression analysis for overall survival, STAS-present patients showed no significant differences in the hazards of death (HR: 1.15; 95% CI: 0.56–2.35; *p* = 0.706). In the weighted multivariable Cox regression analysis, STAS-present patients did not show an increased hazard of an OS event compared with STAS-absent patients (HR: 1.19; 95% CI: 0.58–2.44; *p* = 0.628). Age was not significantly associated with OS (HR: 1.02; 95% CI: 0.99–1.05; *p* = 0.231). The female sex was associated with a significantly lower hazard of an OS event compared with the male sex (HR: 0.54; 95% CI: 0.33–0.87; *p* = 0.012). Regarding the surgical group, patients in group 1 showed a higher but non-significant hazard of an OS event compared with group 2 (HR: 1.61; 95% CI: 0.99–2.60; *p* = 0.054), whereas patients in group 3 did not show a significant difference compared with group 2 (HR: 0.53; 95% CI: 0.05–5.17; *p* = 0.582). A pairwise comparison between surgical groups 1 and 3 also showed no statistically significant difference in the hazard of an OS event (HR for group 1 vs. group 3: 3.06; 95% CI: 0.31–30.48; *p* = 0.341). The proportional hazards assumption was satisfied in the IPTW-weighted multivariable DFS analysis, as indicated by the Schoenfeld test (χ^2^ = 2.45, *p* = 0.78).

*(d)*  *Weighted disease-free survival*

In our sample, the weighted incidence rate of the event was nearly the same among STAS-present and STAS-absent patients (0.0093 vs. 0.0092 per person-month, or approximately 0.93 and approximately 0.92 per 100 person-months, respectively). In the weighted univariable Cox regression analysis for DFS, STAS-present patients showed no significant differences in the hazards of a DFS event (HR: 0.99; 95% CI: 0.54–1.80; *p* = 0.985). In the weighted multivariable Cox regression analysis, STAS-present patients did not show an increased hazard of a DFS event compared with STAS-absent patients (HR: 1.02; 95% CI: 0.57–1.84; *p* = 0.939). Age was not significantly associated with DFS (HR: 1.01; 95% CI: 0.99–1.04; *p* = 0.157). The female sex was associated with a significantly lower hazard of a DFS event compared with the male sex (HR: 0.68; 95% CI: 0.48–0.97; *p* = 0.034). Regarding the surgical group, patients in group 1 showed a higher but non-significant hazard of a DFS event compared with group 2 (HR: 1.40; 95% CI: 0.95–2.08; *p* = 0.093). Similarly, patients in group 3 showed a higher but non-significant hazard compared with group 2 (HR: 1.67; 95% CI: 0.68–4.10; *p* = 0.262). A pairwise comparison between surgical groups 1 and 3 also showed no statistically significant difference in the hazard of a DFS event (HR: 1.19; 95% CI: 0.47–3.04; *p* = 0.712). The proportional hazards assumption was satisfied in the IPTW-weighted multivariable DFS analysis, as indicated by the Schoenfeld test (χ^2^ = 2.03, *p* = 0.846).

## 4. Discussion

In this study, we observed that STAS is an aggressive histological feature, associated with pN2 nodal involvement, stage IIIA disease, and a micropapillary adenocarcinoma predominant pattern. The latter (stage IIIA disease and micropapillary pattern) remained statistically significant even after multivariable binary logistic regression analysis using STAS as the dependent variable (*p* = 0.0303 and *p* = 0.0213, respectively), despite the wide confidence intervals indicating considerable uncertainty. This aspect may suggest a potential prognostic importance of STAS in patients with resected lung adenocarcinoma in whom a closer postoperative surveillance could be of benefit.

However, no statistically significant correlation was found between STAS and OS or DFS. Indeed, even if it is widely accepted in the literature that STAS may be linked to a worse prognosis [7,18,19,20], our results can be explained by the limited STAS-population enrolled in the study (9.1%), which cannot corroborate the independent prognostic impact by itself.

The true prevalence of STAS-positive lung adenocarcinomas in our cohort is likely underestimated, as a substantial proportion of the cases were diagnosed before 2021 [16] and 2024 [8], when STAS was formally acknowledged by the international scientific community as a prognostically relevant parameter in lung adenocarcinoma and recommended as a histologic descriptor in pathology reporting. A systematic review of archival histological slides according to these updated recommendations could help to clarify the gap between our results and those reported in the literature, where its incidence, regardless of the stage, ranges from 28% to 52% [5] and could improve both the intra- and interobserver reproducibility in the identification of STAS. Moreover, the IPTW method was used to reduce the effects of potential confounding factors between the two groups with regard to the outcomes, by up-weighting each patient in the STAS-present group, considering the inverse of the propensity score. Of note, the female sex was consistently associated with more favorable survival outcomes in our cohort, showing, in both the unadjusted and adjusted analyses, a lower hazard than men for OS and DFS. Although the observational nature of the study does not allow causal interpretations, this finding suggests that sex may represent an important prognostic factor to consider when evaluating the survival outcomes in the STAS-present population. 

Few studies [20,21,22,23] demonstrated that STAS was an unfavorable factor for recurrence and long-term survival in patients with lung adenocarcinoma, and the addition of STAS as a pathological T descriptor to the TNM classification is under consideration to date [15,24].

In particular, even if the prognostic impact of STAS after a lobar resection remains controversial and the role of surgical margins is still debated [20,24,25,26], a recent study with a large cohort reported that both DFS and OS were significantly shorter in STAS-positive patients who underwent lobectomy [24].

Moreover, many studies [3,23,24,25,26,27,28] demonstrated that STAS-positive patients treated with a sub-lobar resection had significantly worse recurrence-free survival and a higher risk of locoregional recurrence than those who underwent lobectomy.

Specifically, Kadota et al. [3] conducted a study on 411 patients with stage 1 lung adenocarcinoma, demonstrating that STAS was an independent risk factor for recurrence after limited resection. Subsequently, these findings were confirmed by several studies highlighting the greater prognostic weight of STAS in patients who underwent a sub-lobar resection compared to those who underwent a lobectomy [23,24,25,26,27,28].

However, a critical limitation in clinical practice is that STAS can only be reliably identified on postoperative histopathological examination, restricting its use in preoperative risk stratification and surgical planning.

In addition, given the small number of patients in each subgroup of this study (only 7 STAS-positive patients underwent sub-lobar resections), the results of the comparison may have a limited statistical power, reducing the precision of some estimates and not allowing us to evaluate the true procedure-specific effects.

Nevertheless, our findings suggest that STAS may serve as an important aggressive histological marker, identifying a subgroup of patients in whom the prognostic implications of the extent of pulmonary resection requires further investigation.

In this context, as suggested by several recent studies that explored radiological and radiomics-based models for STAS prediction [29], our findings corroborate the need to evaluate more reliable preoperative or intraoperative methods able to identify STAS before defining an individualized surgical planning.

Instead, regarding the STAS distribution across different stages of adenocarcinoma, we showed that it was mostly present in the pIIIa stage (25.8%, *p* = 0.003), demonstrating its potential aggressive histological feature and supporting the biological plausibility of STAS as a marker of local tumor dissemination. The trend can be explained by its ability to exhibit an incohesive pattern following the epithelial–mesenchymal transition, facilitating the detachment from primary lung cancers [30,31,32] and leading to an increased locoregional recurrence. However, to date, the exact mechanisms underlying this phenomenon remain incompletely understood, reflecting both the intrinsic tumor biology and its interaction with the extent of pulmonary resection. Other clinicopathological features have been studied in terms of their relationship with STAS, including nodal involvement and an adenocarcinoma micropapillary pattern of growth [15,22,27]. In our study, this finding should be interpreted wisely, as the small number of STAS-positive cases may have resulted in an unstable logistic regression estimate, as reflected by the wide confidence interval observed for the micropapillary pattern (OR 18.4, 95% CI 1.39–461.31). However, evidence from a few studies suggested the micropapillary pattern as a potential pathological correlated factor.

Some limitations should be acknowledged: firstly, its retrospective design and single-institution setting may limit the generalizability of the findings. Secondly, the information on smoking status was not available, limiting our ability to account for its potential influence on survival outcomes. Thirdly, molecular and genetic data were not available for the present analysis. The availability of such information could have enriched the interpretation of our results, allowing a more detailed assessment of the biological profile of tumors with STAS and potentially improving the understanding of its prognostic implications. Moreover, STAS-positive patients were under-represented in our cohort, accounting for less than 10% of the overall sample, which may have limited the statistical power to detect the significant associations with survival outcomes. Moreover, because STAS was diagnosed on postoperative pathological specimens, the present study cannot determine the gold-standard surgical treatment for patients with STAS or if the knowledge of one’s STAS status at the time of surgery would have altered the optimal surgical planning. Further studies incorporating clinical, pathological, lifestyle-related, and molecular variables in larger and more balanced cohorts are needed to better define the role of STAS in patients surgically treated for lung adenocarcinoma. Of note, the multivariable survival models implemented in the present study were deliberately specified using a parsimonious adjustment strategy including only age, sex, and surgical intervention group, in order to limit the model instability given the relatively small proportion of STAS-positive patients and the limited number of survival events. Nevertheless, additional pathological prognostic factors, such as the pathological stage, nodal status, and histological characteristics, may be clinically relevant and should be considered in future studies. We suggest that future research explore models incorporating these variables, while carefully accounting for their interrelationships, as the simultaneous inclusion of multiple closely related pathological features could introduce redundancy rather than clarify the independent association with STAS. Although our approach reduced the model complexity and potential collinearity among pathological variables, the residual confounding from established tumor-related prognostic factors cannot be excluded.

Lastly, we did not specifically assess the relationship between STAS and lymph node metastasis; thus, whether STAS is independently associated with nodal involvement, or reflects other aggressive tumor characteristics, was beyond the main aim of the present study. Future research on STAS should further investigate this topic by incorporating comprehensive pathological data and using multivariable analyses to clarify this relationship and its potential biological implications in clinical practice.

In conclusion, our study provides relevant information on the relationship between STAS and clinicopathological characteristics in surgically treated patients with lung adenocarcinoma, demonstrating its potential aggressive histological feature, due to the significant correlation with stage IIIA disease, and an adenocarcinoma micropapillary pattern. For this reason, STAS may identify a subgroup of patients in whom the prognostic implications should be further investigated and a closer postoperative follow-up could be of benefit. Indeed, these findings confirmed the potential value of STAS as a postoperative marker for risk stratification, suggesting the need for reliable preoperative or intraoperative methods for STAS identification able to define a more individualized surgical decision-making and a potential tailored adjuvant treatment. However, due to the absence of a statistically significant difference between the two groups (STAS-present vs. STAS-absent), both in the unweighted and weight–adjusted analysis, any evaluation of the clinical and prognostic impact of STAS should be interpreted with caution by healthcare professionals and further multicenter prospective studies are needed to clarify its role in risk stratification and surgical management.

## Figures and Tables

**Table 1:** Patient baseline characteristics.

	Before IPTW (Mean, SD)			After IPTW (Mean, SD)		
Variables	STAS-Absent (*n* = 299)	STAS-Present (*n* = 30)	SMD	STAS-Absent (*n* = 299)	STAS-Present (*n* = 30)	SMD
**Age (years)**	68.38 (SD: 8.75)	69.77 (SD: 9.50)	0.151	68.50 (SD: 8.73)	67.55 (SD: 10.49)	0.098
**Sex**			0.043			0.010
Female	0.488 (SD: 0.500)	0.466 (SD: 0.507)		0.486 (SD: 0.501)	0.481 (SD: 0.508)	
**Type of surgery**						
Sub-lobar resection	0.274 (SD: 0.447)	0.233 (SD: 0.430)	0.093	0.271 (SD: 0.445)	0.314 (SD: 0.472)	0.095
Lobectomy	0.712 (SD: 0.453)	0.733 (SD: 0.449)	0.046	0.714 (SD: 0.452)	0.671 (SD: 0.477)	0.091
Pneumonectomy	0.013 (SD: 0.115)	0.033 (SD: 0.182)	0.131	0.015 (SD: 0.122)	0.013 (SD: 0.118)	0.011

SD, standard deviation; IPTW, inverse probability of treatment weight; STAS, spread through air spaces.

**Table 2:** Baseline characteristics of patients according to spread through air spaces status.

Variables	All Patients *n* = 329	STAS-Present *n* = 30	STAS-Absent *n* = 299	*p*-Value
**Age (grouped), *n* (%):**				
≥70	160 (48.63%)	18 (11.25%)	142 (88.75%)	0.191
<70	169 (51.37)	12 (7.10%)	157 (92.90%)	
**Sex, *n* (%):**				
Male	169 (51.37%)	16 (9.47%)	153 (90.53%)	0.821
Female	160 (48.63%)	14 (8.75%)	146 (91.25%)	
**Type of surgery (grouped), *n* (%):**				
Sub-lobar resection	89 (27.05%)	7 (7.87%)	82 (92.13%)	0.674
Lobectomy	235 (71.43%)	22 (9.36%)	213 (90.64%)	
Pneumonectomy	5 (1.52%)	1 (20%)	4 (80.00%)	
**Video-assisted thoracoscopic surgery (VATS), *n* (%)**				
Yes	268 (81.46%)	23 (8.58%)	245 (91.42%)	0.479
No	61 (18.54%)	7 (11.47%)	54 (88.52%)	
**Lymphonodal involvement (pN stage classification), *n* (%):**				
pN0	221 (67.17)	23 (10.41%)	198 (89.59%)	0.245
pN1	20 (6.08%)	0 (0.00%)	20 (100%)	-
pN2	22 (6.69%)	5 (22.73%)	17 (77.27%)	**0.022**
pNx	64 (19.45%)	2 (3.13%)	62 (96.87%)	0.063
**Tumor size, grouped *n* (%):**				
(Tis)	1 (0.30%)	0 (0.00%)	1 (100.00%)	-
1 (pT1a; pT1b; pT1c; pT1mi)	173 (52.58%)	11 (6.36%)	162 (93.64%)	0.091
2 (pT2; pT2a; pT2b)	111 (33.74%)	12 (10.81%)	99 (89.19%)	0.376
3 (pT3; pT4)	43 (13.07%)	6 (13.95%)	37 (86.05%)	0.376
4 (pTx)	1 (0.30%)	1 (100%)	0 (0.00%)	-
**Pathological stage (grouped), *n* (%):**				
Early stage (1-Ia, 2-Ib, 3- IIa)	189 (57.45%)	16 (8.47%)	173 (91.53%)	0.115
Advanced stage (4-IIb, 5-IIIa, 6 IIIb)	73 (22.18%)	11 (15.07%)	62 (84.93%)	
**Grading (grouped), *n* (%):**				
Grade 1 or 2	38 (11.55%)	1 (2.63%)	37 (97.37%)	0.135
Grade 3	22 (6.69%)	3 (13.63%)	19 (86.36%)	
**HISTOLOGIC PATTERN**				
**Lepidic pattern, *n* (%):**				
Yes	9 (2.73%)	1 (11.11%)	8 (88.89%)	0.811
No	295 (89.66%)	26 (8.81%)	269 (98.19%)	
**Solid pattern, *n* (%)**				
Yes	58 (17.63%)	3 (5.17%)	55 (94.83%)	0.270
No	246 (74.77%)	24 (9.76%)	222 (90.24%)	
**Macropapillary pattern, *n* (%)**				
Yes	33 (10.03%)	5 (15.15%)	28 (85.85%)	0.180
No	271 (82.37%)	249 (91.88%)	22 (8.12%)	
**Micropapillary pattern, *n* (%)**				
Yes	3 (0.92%)	2 (66.67%)	1 (33.33%)	**<0.001**
No	301 (91.49%)	25 (8.30%)	276 (91.70%)	
**Acinar pattern, *n* (%)**				
Yes	201 (61.09%)	16 (7.96%)	185 (92.04%)	0.430
No	103 (31.30%)	11 (10.68%)	92 (89.32%)	
**INVASIONS**				
**Lymphovascular invasion, *n* (%):**				
Yes	19 (5.77%)	3 (15.79%)	16 (84.21%)	0.521
No	112 (34.04%)	12 (10.71%)	100 (89.29%)	
**Perineural invasion, *n* (%):**				
Yes	2 (0.61%)	0 (0.00%)	2 (100.00%)	0.608
No	129 (39.21%)	15 (11.63%)	114 (34.65%)	
**Pleural invasion, *n* (%)**				
Yes	103 (31.31%)	11 (10.68%)	92 (89.32%)	0.595
No	28 (8.52%)	4 (14.29%)	24 (85.71%)	
**Vascular invasion, *n* (%)**				
Yes	5 (1.52%)	1 (20.00%)	4 (80.00%)	0.551
No	124 (37.69%)	14 (11.29%)	110 (88.71%)	

Values in bold are statistically significant; STAS, spread through air spaces.

**Table 3:** Multivariable binary logistic regression analysis with spread through air spaces as the dependent variable.

Variables	Multivariable Analysis	
	OR (95% CI)	*p*-Value
Intercept	0.049 (0.001–1.954)	
Age (continuous variable)	1.01 (0.95–1.07)	0.7999
Sex (female versus male)	1.25 (0.51–3.08)	0.6262
Micropapillary pattern (yes versus no)	18.40 (1.39–461.31)	**0.0303**
IIIa stage (yes versus no)	5.27 (1.15–20.90)	**0.0213**
pN2 (yes versus no)	0.25 (0.03–1.69)	0.1832

Values in bold are statistically significant; CI, confidence interval; OR, odds ratio.

**Table 4:** Unweighted overall and disease-free survival of the total sample, by spread through air spaces status at different timepoints.

	**All Patients**	**Overall Survival (OS)**							
	***n* = 329**	**Events** **(*n* = 76)**	**Person’s Time at Risk** **(Months)**	**Incidence Rate (Per 100 Person-Months)**	**Median OS, Months** **(NR If Not Reached)**	**12 Months**	**24 Months**	**36 Months**	**60 Months**
**STAS (absent)**	299 (90.88%)	67 (88.16%)	13.43	0.00499 (≃0.50 per 100 person-months)	NR	0.96	0.90	0.83	0.72
**STAS (present)**	30 (9.12%)	9 (11.84)	1362	0.0066076 (≃0.66 per 100 person-months)	NR	0.97	0.83	0.75	0.75
	**All Patients**	**Disease-Free Survival (DFS)**							
	***n* = 329**	**Events** **(*n* = 128)**	**Person Time at Risk (Months)**	**Incidence Rate** **(per 100 Person-Months)**	**Median DFS, Months** **(NR If Not Reached)**	**12 Months**	**24 Months**	**36 Months**	**60 Months**
**STAS (absent)**	299 (90.88%)	114 (115.57%)	122, 379	0.0093 (≃0.93 per 100 person-months)	70.86	0.92	0.81	0.73	0.58
**STAS (present)**	30 (9.12%)	14 (12.43%)	1316	0.0106 (≃1.06 per 100 person-months)	70.70	0.90	0.77	0.69	0.58

STAS, spread through air spaces; OS, overall survival; DFS, disease-free survival; NR, not reported.

**Table 5:** Weighted overall and disease-free survival of the total sample, by spread through air spaces status at different timepoints.

	**All Patients**	**OS**							
	***n* = 329**	**Events** **(*n* = 76)**	**Person’s Time at Risk** **(Months)**	**Incidence Rate** **(per 100 Person-Months)**	**Median OS, Months** **(NR If Not Reached)**	**12 Months**	**24 Months**	**36 Months**	**60 Months**
**STAS (absent)**	299 (90.88%)	67 (88.16%)	13,424	0.0049 (≃0.49 per 100 person-months)	NR	0.96	0.90	0.83	0.72
**STAS (present)**	30 (9.12%)	9 (11.84)	1432	0.0057 (≃0.57 per 100 person-months)	NR	0.97	0.87	0.79	0.79
	**All Patients**	**DFS**							
	***n* = 329**	**Weighted Events** **(*n* = 127.08)**	**Person Time at Risk** **(Months)**	**Weighted Incidence Rate** **(per 100 Person-Months)**	**Median DFS, Months** **(NR If Not Reached)**	**12 Months**	**24 Months**	**36 Months**	**60 Months**
**STAS (absent)**	299 (90.88%)	114.14	12,377	0.0092 (≃0.92 per 100 person-months)	70.86	0.92	0.81	0.73	0.56
**STAS (present)**	30 (9.12%)	12.94	1389	0.0093 (≃0.93 per 100 person-months)	70.70	0.90	0.80	0.75	0.75

STAS, spread through air spaces; OS, overall survival; DFS, disease-free survival; NR, not reported.

## Data Availability

The original contributions presented in this study are included in the article/Supplementary Materials. Further inquiries can be directed to the corresponding author.

## Supplementary Materials

The following supporting information can be downloaded at: https://www.mdpi.com/article/10.3390/diagnostics16172747/s1, Figure S1: Overall survival according to STAS status of the study population at 12, 24, 36, and 60 months; Figure S2: Disease-free survival according to STAS status of the study population at 12, 24, 36, and 60 months; Figure S3: IPTW-balanced overall survival according to STAS status of the study population at 12, 24, 36, and 60 months; Figure S4: IPTW-balanced disease-free survival according to STAS status of the study population at 12, 24, 36, and 60 months; Table S1: Results from univariable Cox regression models according to STAS status; Table S2: Results from multivariable Cox regression models (weighted and unweighted). Table S3. Detailed demographic, surgical, and pathological characteristics of the study population.
