# Supplementary material for: Prognostic Implications of Tumor Spread Through Air Spaces in Patients with Resected Lung Adenocarcinoma
Source: Diagnostics (Basel). 2026 Aug 27;16(17):2747. doi: 10.3390/diagnostics16172747 (PMC13564527; doi:10.3390/diagnostics16172747)
Supplement: Supplementary file 1 [file diagnostics-16-02747-s001.zip › diagnostics-4484271-supplementary.pdf]

Supplementary Materials

Description

The supplementary material includes four Kaplan–Meier survival curves and two tables reporting Cox regression analyses. Specifically, **Figure S1** and **Figure S2** show overall survival and disease-free survival according to STAS status in the study population at 12, 24, 36, and 60 months. **Figure S3** and **Figure S4** presents the corresponding IPTW-balanced survival curves for overall survival and disease-free survival, respectively, according to STAS status at the same time points. For each figure, blue lines denote STAS-absent patients, while red lines denote STAS-present patients. The X-axis represents the number of months elapsed since surgery.

Supplementary Table S1 reports the results of the univariable Cox regression models, while **Supplementary Table S2** summarizes the findings from the multivariable Cox regression models, including both weighted and unweighted analyses.

Supplementary Table S3 reports detailed demographic, surgical, and pathological characteristics of the study population.

Figure S1: Overall survival according to STAS status of the study population at 12, 24, 36, and 60 months.

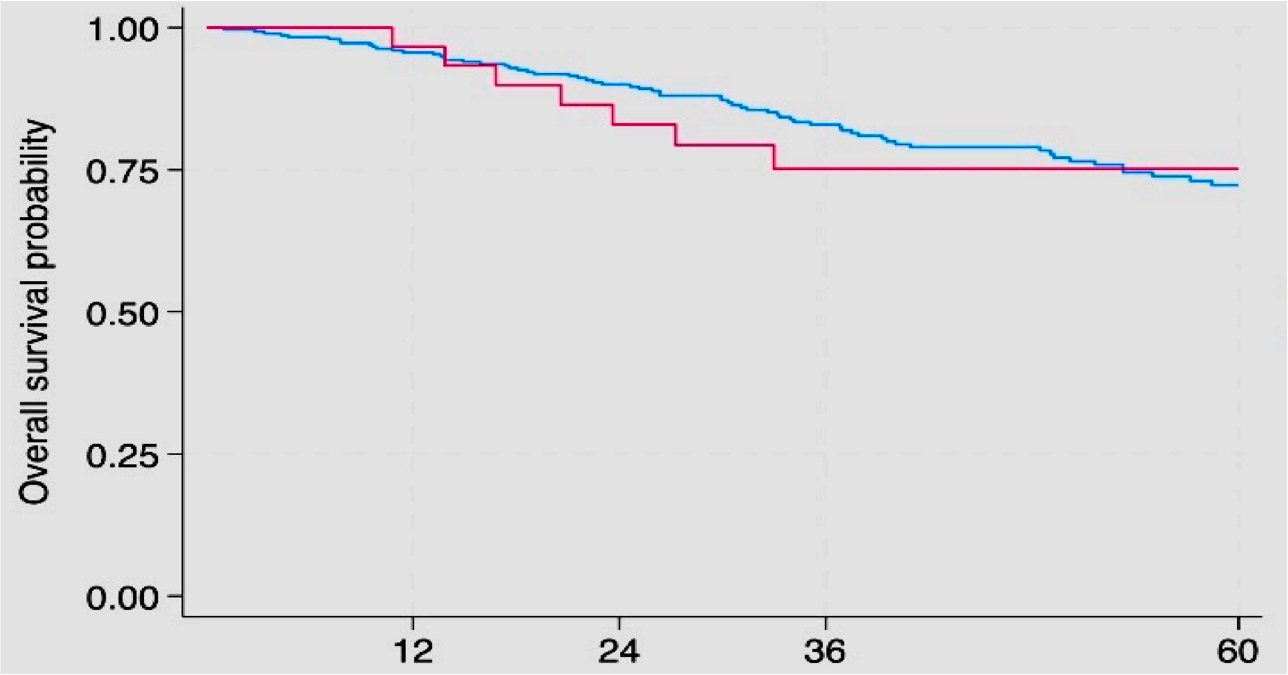

**Number at risk**	**12 months**	**24 months**	**36 months**	**60 months**
STAS-absent	286	235	179	89
STAS-present	29	24	18	9

Figure S2: Disease-free survival according to STAS status of the study population at 12, 24, 36, and 60 months

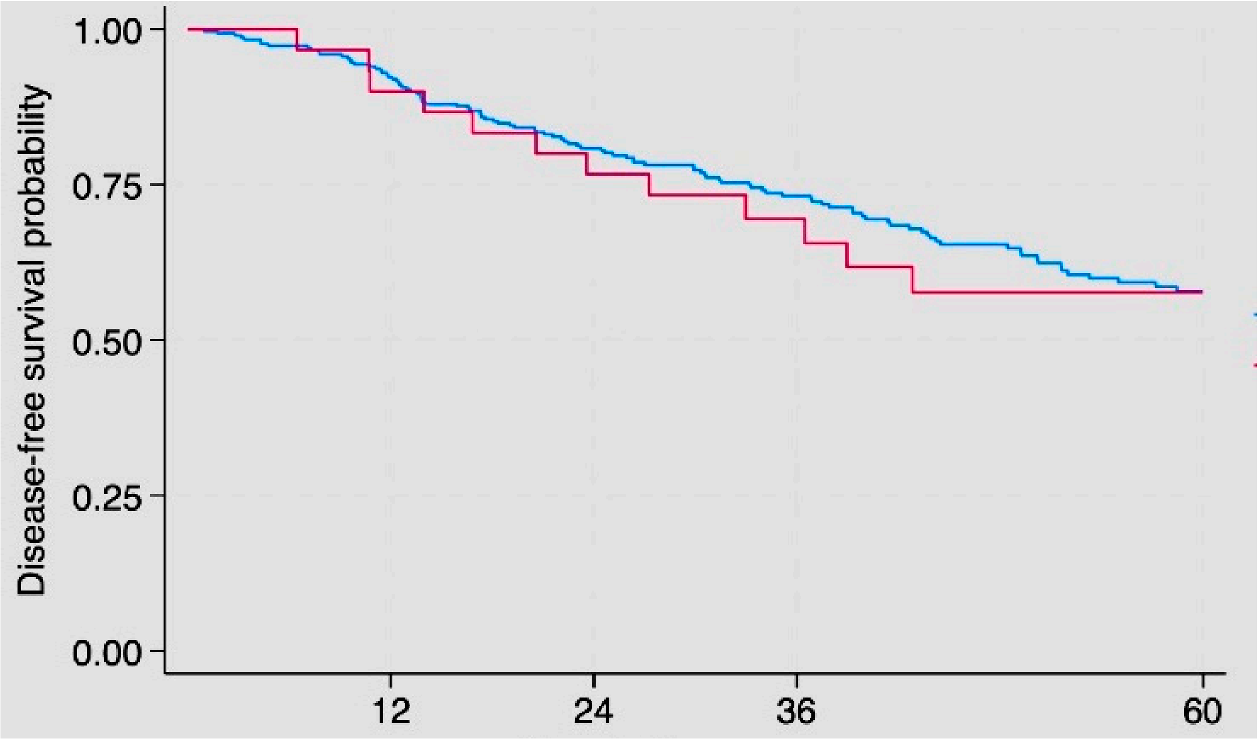

Number at risk	12 months	24 months	36 months	60 months
STAS-absent	276	214	164	78
STAS-present	27	23	18	9

Figure S3: IPTW-balanced overall survival according to STAS status of the study population at 12, 24, 36, and 60 months.

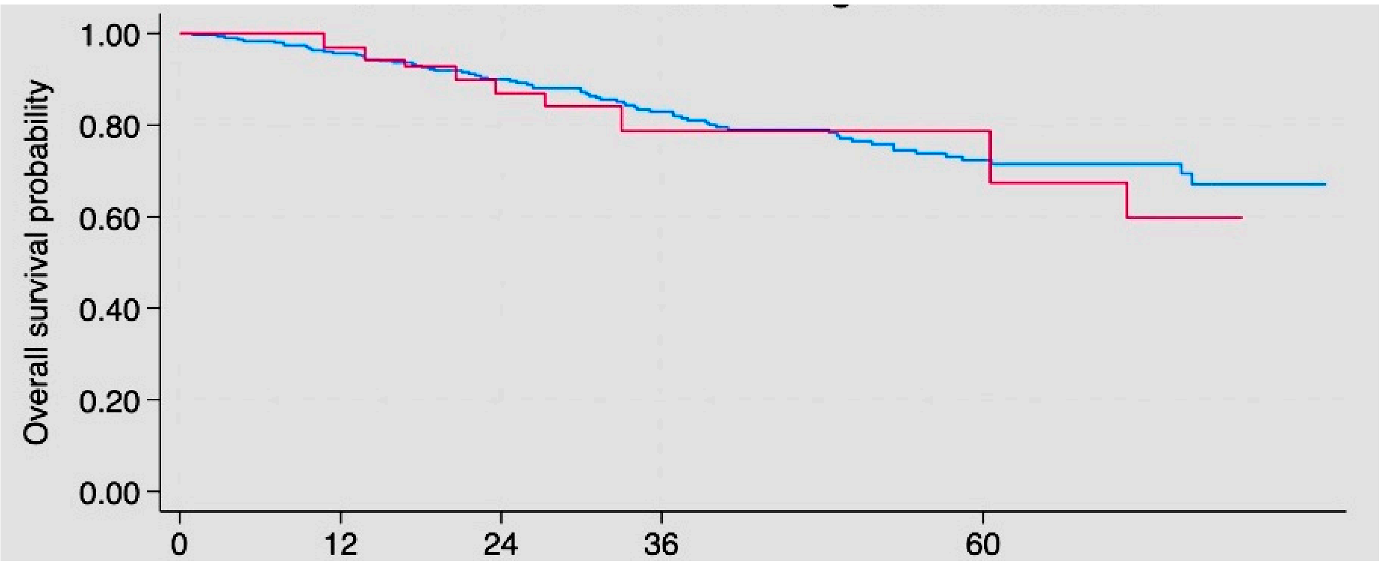

Number at risk	Baseline	12 months	24 months	36 months	60 months
STAS-absent	298.98	286.06	234.93	178.91	89.05
STAS-present	30.40	29.47	25.36	19.11	10.17

Figure S4: IPTW-balanced disease-free survival according to STAS status of the study population at 12, 24, 36, and 60 months.

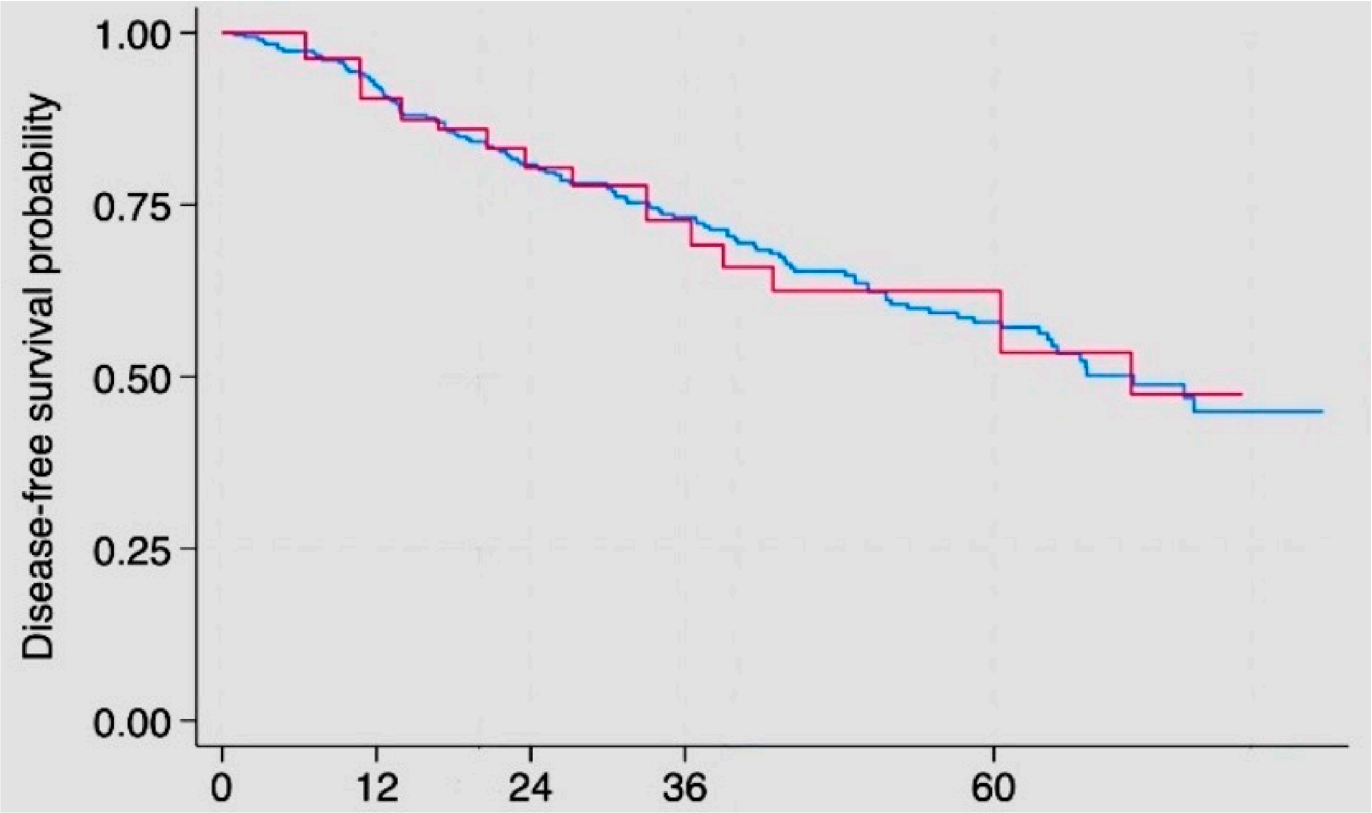

Number at risk	Baseline	12 months	24 months	36 months	60 months
STAS-absent	276.11	213.87	234.93	163.89	78.06
STAS-present	27.49	24.42	25.36	19.11	10.17

Supplementary Table S1. Results from univariable Cox regression models according to STAS status**.**

**Endpoint**	**Weighting**	**Variable**	**HR**	**95% CI**	**p-value**
OS	Unweighted	STAS-absent	1.00	Reference	—
OS	Unweighted	STAS-present	1.33	0.67–2.65	0.415
DFS	Unweighted	STAS-absent	1.00	Reference	—
DFS	Unweighted	STAS-present	1.14	0.65–2.00	0.643
OS	Weighted	STAS-absent	1.00	Reference	—
OS	Weighted	STAS-present	1.15	0.56–2.35	0.706
DFS	Weighted	STAS-absent	1.00	Reference	—
DFS	Weighted	STAS-present	0.99	0.54–1.80	0.985

Supplementary Table S2. Results from multivariable Cox regression models (weighted and unweighted)

**Endpoint**	**Weighting**	**Comparison**	**Reference**	**HR**	**95% CI**	**P value**
OS	Unweighted	STAS-present	STAS-absent	1.37	0.69– 2.71	0.372
OS	Unweighted	Age		1.02	0.99–1.05	0.218
OS	Unweighted	Female sex	Male sex	0.55	0.34–0.89	**0.014**
OS	Unweighted	Sublobar resection	LOBECTOMY	1.64	1.01–2.65	**0.044**
OS	Unweighted	PNEUMONECTOMY	LOBECTOMY	1.39	0.16–12.18	0.766
OS	Unweighted	Sublobar resection	PNEUMONECTOMY	1.18	0.16–8.86	0.872
DFS	Unweighted	STAS-present	STAS-absent	1.15	0.66–2.00	0.619

DFS	Unweighted	Female sex	Male sex	0.7	0.49–0.99	**0.045**
DFS	Unweighted	Sublobar resection	LOBECTOMY	1.45	0.98–2.14	0.062
DFS	Unweighted	PNEUMONECTOMY	LOBECTOMY	2.03	0.80–5.14	0.137
DFS	Unweighted	Sublobar resection	PNEUMONECTOMY	1.4	0.53–3.66	0.496
OS	Weighted	STAS-present	STAS-absent	1.19	0.58–2.44	0.628
OS	Weighted	Age		1.02	0.99–1.05	0.231
OS	Weighted	Female sex	Male sex	0.54	0.33–0.87	**0.012**
OS	Weighted	Sublobar resection	LOBECTOMY	1.61	0.99–2.60	0.054
OS	Weighted	PNEUMONECTOMY	LOBECTOMY	0.53	0.05–5.17	0.582
OS	Weighted	Sublobar resection	PNEUMONECTOMY	3.06	0.31–30.48	0.341
DFS	Weighted	STAS-present	STAS-absent	1.02	0.57–1.84	0.939
DFS	Weighted	Age		1.01	0.99–1.04	0.157
DFS	Weighted	Female sex	Male sex	0.68	0.48–0.97	**0.034**
DFS	Weighted	Sublobar resection	LOBECTOMY	1.4	0.95–2.08	0.093
DFS	Weighted	PNEUMONECTOMY	LOBECTOMY	1.67	0.68–4.10	0.262
DFS	Weighted	Sublobar resection	PNEUMONECTOMY	1.19	0.47–3.04	0.712

Note: HR: hazard ratio; CI: confidence interval; STAS: spread through air spaces; IPTW: inverse probability of treatment weighting. OS: overall survival; DFS: disease-free survival.

Supplementary Table S3. Detailed demographic, surgical, and pathological characteristics of the study population.

**Variables**	**All patients**	**STAS-present**	**STAS-absent**
	**N = 329**	**N = 30**	**N = 299**

**Age in years, mean (SD)**			
	68.51 (8.82)	69.77 (9.50)	68.38 (8.75)
**Type of surgery n (%):**			
1-Right upper lobectomy	90 (27.36%)	9 (30%)	81 (27.09%)
2-Middle lobectomy	8 (2.43%)	1 (3.33%)	7 (2.34%)
3-Right lower lobectomy	35 (10.64%)	5 (16.67%)	30 (10.03%)
4-Left upper lobectomy	64 (19.45%)	3 (10.00%)	61 (20.40%)
5-Left lower lobectomy	38 (11.55%)	4 (13.33%)	34 (11.37%)
6-Right pneumonectomy	2 (0.60%)	1 (3.33%)	1 (0.33%)
7-Left pneumonectomy	3 (0.91%)	0 (0.00%)	3 (1.00%)
8-Upper-middle bilobectomy	5 (1.52%)	0 (0.00%)	5 (1.67%)
9-Lower-middle bilobectomy	5 (1.52%)	1 (3.33%)	4 (1.34%)
10-Wedge Resection	67 (20.36%)	4 (13.33%)	63 (21.07%)
11-Left trisegmentectomy (S1-2-3)	1 (0.30%)	1 (3.33%)	0 (0.00%)
12-S4+5	3 (0.91%)	0 (0.00%)	3 (1.00%)
13- Right S6	7 (2.13%)	1 (3.33%)	6 (2.01%)
14- Left S6	1 (0.30%)	0 (0.00%)	1 (0.33%)
**Tumour size (T) n (%):**			
Tis	1 (0.30%)	0 (0.00%)	1 (0.33%)
pT1a	38 (11.55%)	2 (6.67%)	36 (12.04%)
pT1b	84 (25.53%)	5 (15.67)	79 (26.42%)

pT1c	47 (14.29%)	4 (13.33%)	43 (14.38%)
pT1mi	4 (1.22%)	0 (0.00%)	4 (1.34%)
pT2	50 (15.20%)	7 (23.33%)	43 (14.38%)
pT2a	52 (15.81%)	4 (13.33%)	48 (16.05%)
pT2b	9 (2.74%)	1 (3.33%)	8 (2.68%)
pT3	31 (9.42%)	3 (10.0%)	28 (9.36%)
pT4	12 (3.65%)	3 (10.0%)	9 (3.01%)
pTx	1 (0.30%)	1 (3.33%)	0 (0.00%)
**Pathological Stage, n (%):**			
Ia	107 (32.53%)	7 (6.54%)	100 (93.46%)
Ib	75 (22.80%)	9 (12.00%)	66 (88.00%)
IIa	7 (2.13%)	0 (0.00%)	7 (100.00%)
IIb	38 (11.55%)	3 (7.90%)	35 (92.10%)
IIIa	31 (9.42%)	8 (25.81%)	23 (74.19%)
IIIb	4 (1.21%)	0 (0.00%)	4 (100.00%)
**Grading, n (%):**			
Grade 1	5 (1.52%)	1 (20%)	4 (80%)
Grade 2	33 (10.03%)	-	33 (100%)
Grade 3	22 (6.69%)	3 (13.64%)	19 (86.36%)
